# Outcomes with insulin glargine in patients with type 2 diabetes previously on NPH insulin: evidence from clinical practice in Spain

**DOI:** 10.1111/j.1742-1241.2011.02880.x

**Published:** 2012-02-16

**Authors:** E Delgado, on behalf of LAUREL Spain study investigators

**Affiliations:** Hospital Universitario Central de AsturiasOviedo, Spain

## Abstract

**Aim:**

We evaluated the effectiveness of insulin glargine (glargine)-based regimens in patients with type 2 diabetes mellitus (T2DM) in clinical practice in Spain.

**Methods:**

This was a retrospective, registry-based study of 1482 patients treated with neutral protamine Hagedorn (NPH) who were either switched to glargine or maintained on NPH at investigators’ discretion. The primary outcomes were HbA_1c_ change over a period of 4–9 months follow-up and incidence of hypoglycaemia.

**Results:**

Prior to switching treatment, mean ± standard deviation HbA_1c_ was worse in the glargine vs. the NPH group (8.3 ± 1.2% vs. 7.9 ± 1.1% respectively; p < 0.0001). After 4–9 months of treatment, mean reductions in HbA_1c_ were greater with glargine vs. NPH (−1.0 ± 1.0% vs. −0.2 ± 0.8% respectively; p < 0.0001) and the incidence of hypoglycaemia in the month prior to the study visit was lower (21.8% vs. 47.6% respectively; p < 0.0001). An expected reduction in dosing frequency, as well as in the basal insulin dose was reported for glargine vs. NPH, with 97.3% of glargine-treated patients on once-daily injections and 81.2% on NPH receiving twice-daily therapy. Improvements in treatment satisfaction were significantly higher with glargine (p < 0.0001).

**Conclusions:**

In a Spanish clinical practice setting, patients with T2DM who switched to glargine from NPH experienced significantly greater reductions in mean HbA_1c_ and a lower incidence of hypoglycaemia than patients maintained on NPH.

**What’s known**

A large number of randomised controlled trials (RCTs) have established insulin glargine as an important therapeutic option that has a number of advantages over human insulin preparations, such as neutral protamine Hagedorn (NPH) insulin, in patients with type 2 diabetes, including a lower risk of hypoglycaemia. However, the highly controlled conditions of an RCT are not always reflective of clinical practice and results may not translate directly.

**What’s new**

This report provides evidence from an observational trial that evaluated the benefits of insulin glargine and NPH insulin in clinical practice in Spain. The data provide verification of the clinical benefits of insulin glargine compared with NPH insulin, previously identified in RCTs.

## Introduction

The management of diabetes relies on a critical balance between achieving target glycaemic control [haemoglobin A1c (HbA_1c_)] and avoiding hypoglycaemia (1). In patients with type 2 diabetes mellitus (T2DM), chronic hyperglycaemia has been shown to be strongly linked with an increased risk of micro- and macrovascular complications, such as retinopathy, nephropathy and cardiovascular disease (2,3). Reductions in HbA_1c_ can reduce the risk of complications associated with diabetes (3,4). Indeed, international guidelines call for the achievement and maintenance of strict glycaemic control, while avoiding hypoglycaemia (1). However, the consequences of very intensive glucose control were demonstrated in the ACCORD study, where patients in the intensive group were treated to achieve an HbA_1c_ target of <6.0% (5). Patients who received intensive therapy were at a higher risk of developing severe hypoglycaemia compared with those treated in the conventional arm. Moreover, patients with severe hypoglycaemia had a higher incidence of cardiovascular death compared with patients without severe hypoglycaemia, and although a *post hoc* analysis failed to demonstrate that symptomatic, severe hypoglycaemia explained the excess mortality in the intensive treatment arm (6).

Long-acting insulin analogues have been shown to provide consistent glycaemic control with a lower incidence of hypoglycaemia compared with conventional insulin treatment with neutral protamine Hagedorn (NPH) insulin (7–10). In head-to-head randomised controlled trials (RCTs) of patients with T2DM, insulin glargine and NPH insulin resulted in similar glycaemic control (including HbA_1c_), although insulin glargine was associated with a significantly reduced risk of hypoglycaemia compared with NPH insulin (11–13). However, RCTs may not always reflect the reality of day-to-day management, as these trials are performed under strict control with stringent patient inclusion/exclusion criteria. Therefore, we designed the present registry to reflect the management of patients in clinical practice with T2DM treated with basal insulin in Spain. We also aimed to gain a realistic perspective on the balance between glycaemic control and hypoglycaemia achieved with insulin glargine and NPH insulin in this population.

## Research design and methods

### Objectives

The main objective of the LAUREL (LANTUS Utilization in REal Life) Spain study was to evaluate outcomes for patients with T2DM switching from an NPH- to an insulin glargine-based regimen with patients continuing on NPH insulin as control group in a Spanish clinical practice setting.

### Study design

**Figure 1:**
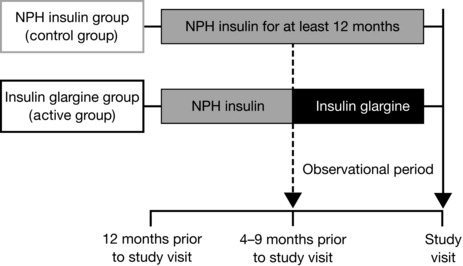
Design of the LAUREL (LANTUS Utilization in REal Life) – Spain study. NPH, neutral protamine Hagedorn

This was a retrospective, registry-based study conducted within everyday clinical practice in Spain. The study consisted of a single visit, during which eligible patients were identified and data for the preceding 12 months of treatment were collected and analysed (Figure 1). The study population consisted of two groups. The first group comprised those who remained on NPH insulin for at least 12 months, whereas the second group were those who switched from NPH insulin to insulin glargine 4–9 months prior to the study visit. All treatment decisions, including the decision to switch insulins, dosing and titration, were at the discretion of the treating investigator/physician. Each investigator recruited six consecutive patients who fulfilled all the selection criteria (four patients for the insulin glargine group and two patients for the NPH insulin group). The study was approved by an independent ethics committee at the Hospital Universitario Central de Asturias (HUCA, Oviedo, Spain).

### Study population

Men and women (>18 years) with T2DM were included. The insulin glargine group consisted of patients whose treating physician decided to switch the patient from NPH insulin to insulin glargine during a 4–9 month period prior to inclusion. Patients in the NPH insulin group were those who were maintained on NPH insulin for at least 12 months. The choice of additional specific medications, such as rapid-acting insulins and oral antidiabetic drugs (OADs), was also at the discretion of the treating physician. All patients provided written consent prior to study entry.

Patients were excluded if they were receiving any form of intermediate- or long-acting insulin other than NPH insulin or insulin glargine (e.g. insulin detemir) or fixed mixtures of long- and short-acting insulin (regular human insulin or analogue); or patients with significant modifications to their rapid-acting insulin during the 6 months prior to inclusion in the study. Changes to OAD regimens were permitted. Women who were pregnant, lactating or had gestational diabetes and patients with any clinically significant acute major organ or systemic diseases were also excluded.

Contributing investigators were endocrinologists or internists specialising in insulin prescription. Patients and investigators were identified by codes on the clinical report forms to maintain confidentiality, and a further internal database code was assigned by the contract research company responsible.

### Study assessments

Retrospective data from the 4–9 month observational period prior to and including the study visit were collected for HbA_1c_, fasting blood glucose (FBG), insulin and OAD treatment. In the group treated with insulin glargine, this information was collected at the time of the change from NPH to insulin glargine, which took place during the 4–9 month period prior to the study visit. For the NPH insulin group, this information was collected at a visit conducted during an equivalent 4–9 month window preceding the study visit. Data were adjusted for time since collection as part of the statistical analysis.

Documented hypoglycaemia episodes (severe and non-severe) recorded during the month prior to the study visit and self-monitored blood glucose readings were collected at the study visit. Hypoglycaemia was defined using the following criteria: symptomatic, symptoms of hypoglycaemia accompanied by a measured plasma glucose (PG) level of ≤ 70 mg/dl (3.9 mmol/l); asymptomatic, a measured PG level of ≤ 70 mg/dl (3.9 mmol/l); nocturnal, an event that occurred while the patient was asleep, after bedtime and before getting up in the morning, accompanied by a PG level of ≤ 70 mg/dl (3.9 mmol/l).

Treatment satisfaction was measured using the validated Diabetes Treatment Satisfaction Questionnaire change (DTSQc) and status (DTSQs) questionnaires, which were completed by patients at the study visit (14,15). The DTSQc asked patients in the insulin glargine group to assess any change in their experience over the observational period vs. their previous experience with NPH insulin. Patients in the NPH insulin group were asked to assess any change in their experience with NPH insulin over the observational period. Patients also completed the DTSQs, which asked them to recall their experience with NPH insulin during the last few weeks of treatment prior to changing to insulin glargine (insulin glargine group) or before the period 4–9 months prior to the study visit (NPH insulin group). Maximum scores of 18.00 (DTSQc) and 36.00 (DTSQs) indicated improvement in treatment satisfaction and optimal treatment satisfaction. For the six questions that assess treatment satisfaction, each item was scored from +3 (much more satisfied now) to –3 (much less satisfied now) in the DTSQc and from 6 (very satisfied) to 0 (very dissatisfied) in the DTSQs. The perceived frequency of hyper- and hypoglycaemia scores ranged from +3 (DTSQc) and 6 (DTSQs) (most of the time) to –3 (DTSQc) and 0 (DTSQs) (none of the time) respectively.

### Study endpoints

The primary endpoints were the change in HbA_1c_ over the observational period from the time of the insulin switch (4–9 months prior to study visit) to the time of inclusion (study visit), and the incidence of hypoglycaemia (severe and non-severe) in the month prior to the study visit in the insulin glargine and NPH insulin groups. Secondary endpoints included changes in FBG, change in dose and dosing frequency of basal insulin and treatment satisfaction as measured using the DTSQc and DTSQs questionnaires.

### Statistical analysis

The difference in HbA_1c_ change between the insulin glargine arm and the NPH insulin arm was estimated to be 0.2% in favour of insulin glargine. With an alpha risk (two-sided test) of 5%, 80% power, a standard deviation (SD) of 1.1% and a non-evaluable rate of 10%, the total number of patients to be included was 523 in each treatment group. Previous studies of insulin glargine in patients with T2DM have found the incidence of severe hypoglycaemia to be approximately 2.5% (12). It was estimated that a sample size of 858 patients was needed in the insulin glargine arm of this study to be able to detect a documented severe hypoglycaemia rate of 2.5% with a precision of ± 1% and a confidence interval (CI) of 95%. Based on this calculation, investigators had to recruit approximately two insulin glargine-treated patients for every one NPH insulin-treated patient, until 1416 patients were recruited (NPH insulin, *n* = 472; insulin glargine, *n* = 944, considering a non-evaluable rate of 10%). Despite the reduction in sample size for the NPH insulin group (523 to 472 patients), the recruited global sample enabled detection of a difference of 0.2% (SD 1.1%) in the mean change in HbA_1c_ between the treatment groups. Therefore, each investigator included four patients treated with insulin glargine and two patients treated with NPH insulin.

Data were presented as means ± standard deviation, percentage or median (minimum–maximum) as appropriate. All data were analysed using SPSS Version 14 statistical software (SPSS Inc., Chicago, IL, USA). Confidence intervals were calculated at 95%. Comparisons were made on two-tailed tests and considered significant when p < 0.05. Student’s paired *t-*test was used to compare parameters before and after the treatment period for independent samples. A multivariate analysis of covariance was used to adjust outcome variables with statistically significant baseline differences across treatment groups. Adjustments were made for age, gender, body mass index (BMI), concomitant diseases and comorbidities, previous HbA_1c_ value, time since HbA_1c_ assessment, FBG value, time since diabetes diagnosis (years), time since insulinisation, changes in treatment with rapid-acting insulin and OADs, number of injections per day and insulin doses.

## Results

### Study population and baseline characteristics

A total of 1662 patients were enrolled; of these, 180 (10.8%) were non-evaluable, mainly because they did not meet the inclusion criteria of treatment with insulin glargine (*n* = 133) or NPH insulin (*n* = 13). Four patients (0.2%) had no data for the analysis of the primary objective, treatment group was not recorded for 29 patients (1.7%) and one patient (0.1%) had type 1 diabetes. Therefore, 1482 patients were included in the analyses: 976 (65.9%) patients in the insulin glargine group [465/483 men/women; mean ± SD age of 61.9 ± 11.0 years] and 506 (34.1%) patients in the NPH insulin group (225/276 men/women; mean ± SD age of 64.3 ± 11.4 years) respectively. Patient demographics and clinical characteristics at study visit are presented in Table 1.

**Table 1:** Patient demographics and clinical characteristics at study visit

	Insulin glargine (*n* = 976)	NPH insulin (*n* = 506)
Age, years*	61.9 ± 11.0	64.3 ± 11.4
Male/female, n†	465/483	225/276
Weight, kg‡	77.9 ± 12.3	76.8 ± 12.1
BMI, kg/m^2^§	29.0 ± 4.3	29.0 ± 4.2
HbA_1c_	7.3 ± 0.9	7.8 ± 1.1
Systolic blood pressure, mmHg¶	138.4 ± 15.6	138.6 ± 17.6
Diastolic blood pressure, mmHg**	80.0 ± 10.4	79.9 ± 10.3
Time since diabetes diagnosis, years††	10.2 (0.5–49)	12.4 (1.3–42)
Time since insulinisation, years‡‡	4 (0.25–49)	5 (1–31)
Time since current insulin treatment§§	5.3 (3–10) months	5 (1–31) years

Data are presented as means ± standard deviation, percentage or median (minimum–maximum), as appropriate. Missing data (*n*) for insulin glargine/NPH insulin: *8/2; †28/5; ‡6/3; §8/2; ¶13/10; **14/10; ††14/6; ‡‡7/4; §§174/38. NPH, neutral protamine Hagedorn; BMI, body mass index.

### Glycaemic control

**Figure 2:**
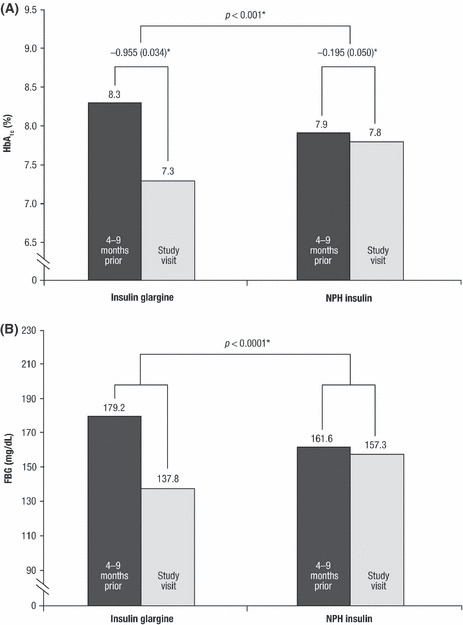
Glycaemic control. (A) Change in glycosylated haemoglobin A_1c_ from 4 months to 9 months prior to inclusion to study visit. *Change in HbA_1c_ (standard error) and p value (analysis of covariance) adjusted for confounding factors; Missing data (*n*) for insulin glargine/NPH insulin groups: 273/182; NPH, neutral protamine Hagedorn. (B) Change in fasting blood glucose from 4 months to 9 months prior to inclusion to study visit. *Student’s *t-*test; NPH, neutral protamine Hagedorn; FBG=fasting blood glucose

The mean ± SD time between HbA_1c_ assessments for the insulin glargine and the NPH insulin groups was 5.7 ± 1.5 and 5.3 ± 1.5 months respectively. Prior to switching treatment, the mean HbA_1c_ level in patients whose treating physician had switched them to insulin glargine was significantly worse than that of the patients who continued on NPH insulin: mean ± SD, 8.3 ± 1.2% vs. 7.9 ± 1.1% respectively (p < 0.0001; Figure 2A). Nevertheless, the mean change (SD) in HbA_1c_ over the duration of observation was significantly greater in patients in the insulin glargine group compared with the NPH insulin group (−1.0 ± 1.0 vs. −0.2 ± 0.8; p < 0.0001). As a result, the mean HbA_1c_ level at the study end was significantly lower in the insulin glargine group vs. the NPH group: 7.3 ± 0.9% vs. 7.8 ± 1.1% respectively (p < 0.0001). A significant difference in favour of insulin glargine remained after adjustment for the potentially confounding factors of age, gender, BMI, concomitant diseases and comorbidities, previous HbA_1c_ value, time since HbA_1c_ assessment, FBG value, time since diabetes diagnosis, time since insulinization, changes in treatment with rapid-acting insulin and OADs, number of injections per day and insulin dose: mean ± standard error, insulin glargine group −0.874 ± 0.031% and NPH insulin group −0.363 ± 0.047% (p < 0.001, analysis of covariance; Figure 2A).

Patients in the insulin glargine group also had significantly greater reductions in FBG levels over the observational period compared with patients in the NPH insulin group (p < 0.0001), and had lower FBG levels at the study visit (137.8 and 157.3 mg/dl, respectively; Figure 2B).

### Hypoglycaemic events

**Figure 3:**
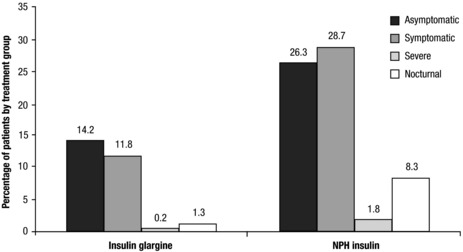
Percentage of patients experiencing at least one hypoglycaemic event in the month prior to study visit. NPH, neutral protamine Hagedorn

At the study visit, 213 patients [21.8% (95% CI: 19.2–24.4%)] in the insulin glargine group and 241 [47.6% (95% CI: 43.3–52.2%)] in the NPH insulin groups had experienced hypoglycaemia (any category) in the previous month (p < 0.0001). The incidence of hypoglycaemia according to severity for both treatment groups is presented in Figure 3. There was a trend towards a higher incidence of all types of hypoglycaemia with NPH insulin, which was most notable for symptomatic hypoglycaemia.

### Changes in basal insulin and oral therapy dose

The median (range) dose of NPH insulin for the insulin glargine group prior to switching treatment was 34 (6–120) IU/day, with 79.0% of patients receiving twice-daily NPH insulin injections. The equivalent median dose for the NPH insulin group was 34 (10–110) IU/day, with 81.8% of patients receiving NPH insulin twice-daily. At the study visit, patients who had switched to insulin glargine had an expected reduction in the frequency of basal insulin injections and in the dose compared with the NPH insulin group, in keeping with insulin glargine prescribing recommendations for once-daily dosing (16). In the insulin glargine group, 97.3% of patients were administering insulin glargine once daily at a median dose of 30 IU/day (range: 6–100 IU/day). In the NPH insulin group, 81.2% of patients were administering NPH insulin twice daily at a median range of 36 IU/day (range: 8–110 IU/day).

At the study visit, patients receiving insulin glargine were less likely than NPH-treated patients to be receiving concomitant metformin or acarbose, but substantially more likely to be receiving repaglinide (Table 2). The pattern of change in oral therapy at the study visit from 4 months to 9 months previous was broadly comparable between the treatment groups.

**Table 2:** Oral antidiabetic therapy

	4–9 months prior				Study visit			
		
	Proportion (%)		Mean dose (mg)		Proportion (%)		Mean dose (mg)	
				
	GLAR	NPH	GLAR	NPH	GLAR	NPH	GLAR	NPH
Metformin	83.6	89.4	1564 ± 599	1531 ± 557	87.3	92.1	1605 ± 547	1552 ± 552
**Meglitinide**	20.7	15.9			31.2	17.3		
Repaglinide	20.4	15.5	5.3 ± 2.4	4.9 ± 1.9	31.2	17.3	5.3 ± 2.4	5.1 ± 2.4
Nateglinide	0.2*	0.4*	–*	–*	–	–	–	–
**α-glucosidase inhibitor**	7.9	8.7			3.2	6.1		
Acarbose	7.9	8.7	173 ± 81	205 ± 82	2.9	5.8	200 ± 92	203 ± 78
Miglitol	–	–	–	–	0.3	0.3	125 ± 35	100 ± 0
**Sulfonylurea**	28.2	25.0			17.6	18.5		
Gliclazide	3.7	1.5	163 ± 90	155 ± 74	2.8	1.2	100 ± 89	125 ± 47
Glipizide	0.7	0.8	15 ± 0	10 ± 7	0.6	0.3	12 ± 6	10 ± 0
Glibenclamide	10.1	8.0	12 ± 4	11 ± 5	2.5	3.3	13 ± 4	12 ± 4
Glipentide	0.3	–	3 ± 1	–	0.3	–	4 ± 0	–
Glimepiride	13.3	14.4	5 ± 2	6 ± 4	11.6	13.4	5 ± 2	6 ± 4
**Glitazone**	2.7	1.5			3.8	3.6		
Pioglitazone	1.9	0.8	28 ± 5	30 ± 0	2.2	2.4	30 ± 9	30 ± 8
Rosiglitazone	0.8	0.8	6 ± 2	6 ± 3	1.5	1.2	5 ± 3	6 ± 2

* One patient in each group was receiving nateglinide, but the dose was not recorded. GLAR, insulin glargine group; NPH, neutral protamine Hagedorn.

### Diabetes treatment satisfaction questionnaire (change version)

**Figure 4:**
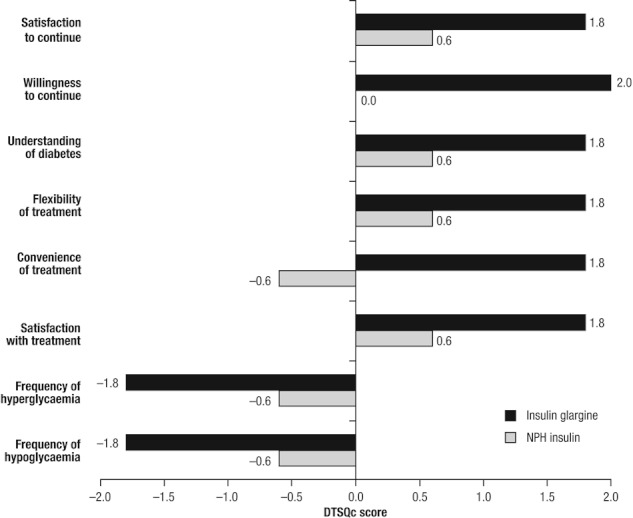
Diabetes Treatment Satisfaction Questionnaire (change version) scores at study visit. Data are presented as median DTSQc scores on a seven-point scale from +3 to −3, with 0 indicating no change. For the first six items shown, a score of +3 represents an improvement and −3 represents a deterioration. In contrast, for the perceived frequency of hyper- and hypoglycaemia items, a score of +3 indicates that events occurred most of the time, indicating a limitation of treatment for patients, whereas a score of −3 indicates no events, suggesting a benefit. DTSQc, Diabetes Treatment Satisfaction Questionnaire (change version); NPH, neutral protamine Hagedorn

Change in overall treatment satisfaction, as measured by the DTSQc scores, was significantly higher (10.2 ± 5.2 vs. 1.4 ± 5.8; p < 0.0001) in insulin glargine-treated patients than in NPH insulin-treated patients at the study visit (Figure 4). In terms of the perception of frequency of hyperglycaemia/hypoglycaemia, patients in the insulin glargine group perceived a significantly greater reduction in the frequency of both hyperglycaemia and hypoglycaemia over the observational period following their switch from NPH insulin, than patients who remained on NPH insulin for the duration of the study (p < 0.0001).

### Diabetes treatment satisfaction questionnaire (status version)

No difference between the two groups was detected in overall treatment satisfaction 4–9 months prior to inclusion in the study (i.e. when all patients were on NPH insulin), as measured using DTSQs score (17.6 ± 7.1 vs. 17.2 ± 6.0; p = 0.343). No difference in the perception of frequency of hypoglycaemia was detected 4–9 months prior to inclusion in the study (2.4 ± 1.5 vs. 2.3 ± 1.3; p = 0.190) between the two treatment groups. However, patients who were subsequently switched to insulin glargine perceived the frequency of hyperglycaemia while still being treated with NPH insulin to be higher compared with patients who subsequently remained on NPH insulin for the duration of the study (3.4 ± 1.5 vs. 2.9 ± 1.2; p < 0.0001).

## Discussion and conclusions

This registry-based study of clinical practice in Spain showed that glycaemic control was significantly improved in patients with T2DM who were switched from NPH insulin to insulin glargine compared with patients maintained on NPH insulin. Fewer patients experienced documented hypoglycaemia with insulin glargine compared with NPH insulin treatment. Furthermore, patients who switched to insulin glargine experienced a reduction in the number of daily basal insulin injections compared with patients who continued on NPH insulin. The clinical benefits of switching to insulin glargine were also associated with improved treatment satisfaction compared with patients maintained on NPH insulin.

While RCTs provide valuable information in terms of the way basal insulin therapy should be initiated in people with T2DM, few studies have compared or confirmed whether such methods are successful in general clinical practice. A number of reports from observational studies in clinical practice have shown improvements in glycaemic control when patients are switched from NPH insulin to insulin glargine (17,18). These data support the findings of our study, which together indicate that insulin glargine may provide greater efficacy in terms of glycaemic control than NPH insulin outside the limits of a controlled trial.

The significant decrease in HbA_1c_ achieved with insulin glargine in a population with long-standing T2DM pretreated with insulin supports the idea that reduced perception of hyper/hypoglycaemia could help to improve glycaemic control in clinical practice. Responses to the DTSQc indicate that patients who switched to insulin glargine perceived significantly greater improvements in the frequency of both hypo- and hyperglycaemia over the observational period than those in the NPH insulin group. Patients in the insulin glargine group also reported greater satisfaction with the flexibility and convenience of treatment, which may reflect the fact that 97.3% in this group received once-daily treatment, compared with more than 80% of patients in the NPH insulin group who received twice-daily injections. All of these findings may reflect the improved glycaemic control reported over the observational period and the lower incidence of hypoglycaemic events in the month prior to the study visit in patients switched to insulin glargine compared with patients who continued on NPH insulin. It is also feasible that patients switching from twice-daily NPH insulin to once-daily insulin glargine may have had improved adherence to the new treatment regimen because of their simplified dosing schedule. That patients in the insulin glargine group reported a significantly higher perceived frequency of hyperglycaemia prior to their switch from NPH insulin, compared with those who remained on NPH insulin for the duration of the study (3.4 ± 1.5 vs. 2.9 ± 1.2; p < 0.0001), could be a reflection of the poor state of their glycaemic control at that point in time, probably triggering the need for their switch to insulin glargine.

The limitations of the retrospective, observational design of this study must be acknowledged. First, patients in this study were not randomised to the treatment groups; instead, the decision for patients to continue NPH insulin or switch to insulin glargine was taken independently by the physicians based on their clinical judgement during a visit performed 4–9 months prior to inclusion in the study. This may have resulted in differences between the groups. The insulin glargine treatment group, for example, had poorer glycaemic control prior to switching treatment compared with the NPH insulin group, which can, in itself, be associated with a greater reduction in HbA_1c_ with intervention. However, in this study, previous HbA_1c_ values were adjusted for in the multivariate analysis. Second, with regard to the evaluation of hypoglycaemic episodes in the month prior to the study visit, because of the retrospective nature of the assessment, there were no recommendations in place for the consistent recording of such events. Thus, findings should be interpreted with caution. Furthermore, as with any study of similar design, the retrospective evaluation of patient-reported outcomes, such as treatment satisfaction, can be subject to a certain amount of recall bias. In the case of this study, the poorer glycaemic control observed in the insulin glargine group prior to the switch from NPH insulin to insulin glargine, and significant improvements vs. the NPH insulin group at the study visit, may have influenced patients’ reporting of historical treatment satisfaction. Finally, factors that may have influenced outcomes, such as hypoglycaemic episodes prior to switching or treatment compliance after switching could not be recorded. Therefore, comparisons between the treatment groups should be interpreted with caution.

Nevertheless, our findings are in line with the results of RCTs in patients with T2DM (11–13,19). Patients in the AT.LANTUS study had a long duration of diabetes (approximately 12 years), and the majority (72%) were already on insulin therapy at baseline (19). The introduction of insulin glargine resulted in an improvement in glycaemic control (as measured by HbA_1c_ and FBG). Of note, a reduction in HbA_1c_ of > 1% was achieved despite the high levels of insulin pretreatment at baseline.

The benefits of glycaemic control in T2DM are well established, and management guidelines provide clear guidance on treatment targets (1–4). The consensus statement from the American Diabetes Association and the European Association for the Study of Diabetes states that a HbA_1c_ level of 7% or more should serve as ‘a call to action to initiate or change therapy with the goal of achieving an HbA_1c_ level of < 7%’. However, this study provides further evidence that these guidelines are not always followed in clinical practice. Patients in the NPH insulin group of this study had a mean HbA_1c_ level of 7.9% at the beginning of the observational period, which fell to 7.8% over the 4–9 month period up to the study visit. In contrast, the mean HbA_1c_ level of 7.3% in the insulin glargine group at the study visit indicates that switching patients who are failing treatment with NPH insulin to insulin glargine may markedly increase the number of patients achieving the recommended target of < 7% while reducing the risks associated with hypoglycaemia. The relative improvements in glycaemic control and hypoglycaemic events with insulin glargine compared with NPH insulin reported in this study are likely to translate to increased overall cost-effectiveness with insulin glargine, which may counter any increase in the direct cost of switching from NPH insulin.

In conclusion, this registry reflects the management of patients in clinical practice with T2DM treated with basal insulin in Spain, and shows that switching patients from an NPH insulin-based regimen to an insulin glargine-based treatment is associated with significant improvements in glycaemic control and incidence of hypoglycaemia.
